# Case report: Successful treatment with daratumumab for pure red cell aplasia in a patient with mixed lymphoid chimerism after ABO-mismatched stem cell transplant for sickle cell disease

**DOI:** 10.3389/fimmu.2023.1212007

**Published:** 2023-06-23

**Authors:** E. Dovern, B. J. Biemond, E. Nur

**Affiliations:** ^1^ Department of Hematology, Amsterdam University Medical Centers, location University of Amsterdam, Amsterdam, Netherlands; ^2^ Department of Blood Cell Research, Sanquin Research and Landsteiner Laboratory, Amsterdam, Netherlands

**Keywords:** sickle cell disease, hematopoietic (stem) cell transplantation (HCST), pure red cell aplasia (PRCA), mixed chimerism, daratumumab (DARA)

## Abstract

Pure red cell aplasia (PRCA) is a serious complication after ABO-mismatched allogeneic hematopoietic stem cell transplantation (HSCT). Following HSCT, persistent anti-donor isohemagglutinins against donor ABO antigens are considered the immunological cause of PRCA. Patients with post-transplant PRCA are at risk for graft rejection and prolonged red blood cell transfusion dependency. No standard treatment exists. Recently however, the anti-CD38 monoclonal antibody daratumumab has been reported to be an effective treatment for post-transplant PRCA in patients with complete donor chimerism. Here, we describe the first case of PRCA in a patient with mixed lymphoid patient/donor chimerism that was successfully treated with daratumumab. This is also the first report of a transplant recipient with sickle cell disease who was treated with this relatively new approach. Fourteen months post-transplantation and twelve months after treatment with daratumumab, our patient has a normal complete blood count and the anti-donor isohemagglutinins remain undetectable despite mixed lymphoid chimerism. Mixed chimerism is a common manifestation in adult patients with sickle cell disease transplanted with non-myeloablative conditioning and a matched sibling donor. The application of non-myeloablative HSCT for patients with sickle cell disease is steadily increasing. Therefore, the incidence of PRCA in this setting might also increase. As the risk of graft rejection due to PRCA can be especially high in patients with mixed chimerism, clinicians should be aware that daratumumab can be an effective treatment in the setting of mixed chimerism.

## Introduction

Allogeneic hematopoietic stem cell transplantation (HSCT) is currently the only established curative treatment option for sickle cell disease (SCD). In adults with SCD, myeloablative conditioning is associated with significant toxicity, primarily because of cumulative organ damage. Newer non-myeloablative conditioning regimens, however, have rendered allogeneic HSCT a viable treatment option for adult SCD patients (1, 2). These conditioning regimens usually result in mixed chimerism.

Pure red cell aplasia (PRCA) after ABO-mismatched HSCT is a serious complication that is associated with delayed engraftment (3, 4). Furthermore, PRCA requires chronic red blood cell (RBC) transfusions, which can result in iron overload and alloimmunization, especially in patients with pre-HSCT alloimmunization. After reduced-intensity conditioning, the incidence of PRCA was reported to be as high as 50% in major ABO-mismatched recipients (5). The persistence of recipient-derived isohemagglutinins against donor ABO antigens is considered the immunological cause of PRCA.

Mixed chimerism following HSCT with a major AB0-mismatched donor in patients with SCD might be associated with an even higher risk of PRCA, as a significant proportion of the adaptive immunity in these patients is still recipient-derived. For this reason, the first studies evaluating non-myeloablative conditioning regimens (alemtuzumab/TBI) in matched sibling donor transplantations in SCD patients excluded patients with AB0-mismatched donors (1). The use of an AB0-mismatched donor was later shown to be feasible, but the exact risk of PRCA and its implications for graft failure in transplanted SCD patients with mixed chimerism is not known.(2) Although pre-transplant rituximab has been used as a strategy to prevent PRCA, it remains unclear whether it significantly decreases its incidence (6).

Recently, several cases of post-transplantation PRCA have been described that were successfully treated with the anti-CD38 monoclonal antibody daratumumab (7, 8). All the described cases had complete donor chimerism. Here, we describe the first case of PRCA in a patient with SCD, undergoing major ABO-mismatched allogeneic HSCT with alemtuzumab/TBI conditioning, resulting in mixed (T-cell) chimerism, who was successfully treated with daratumumab.

## Case description

**Figure 1:**
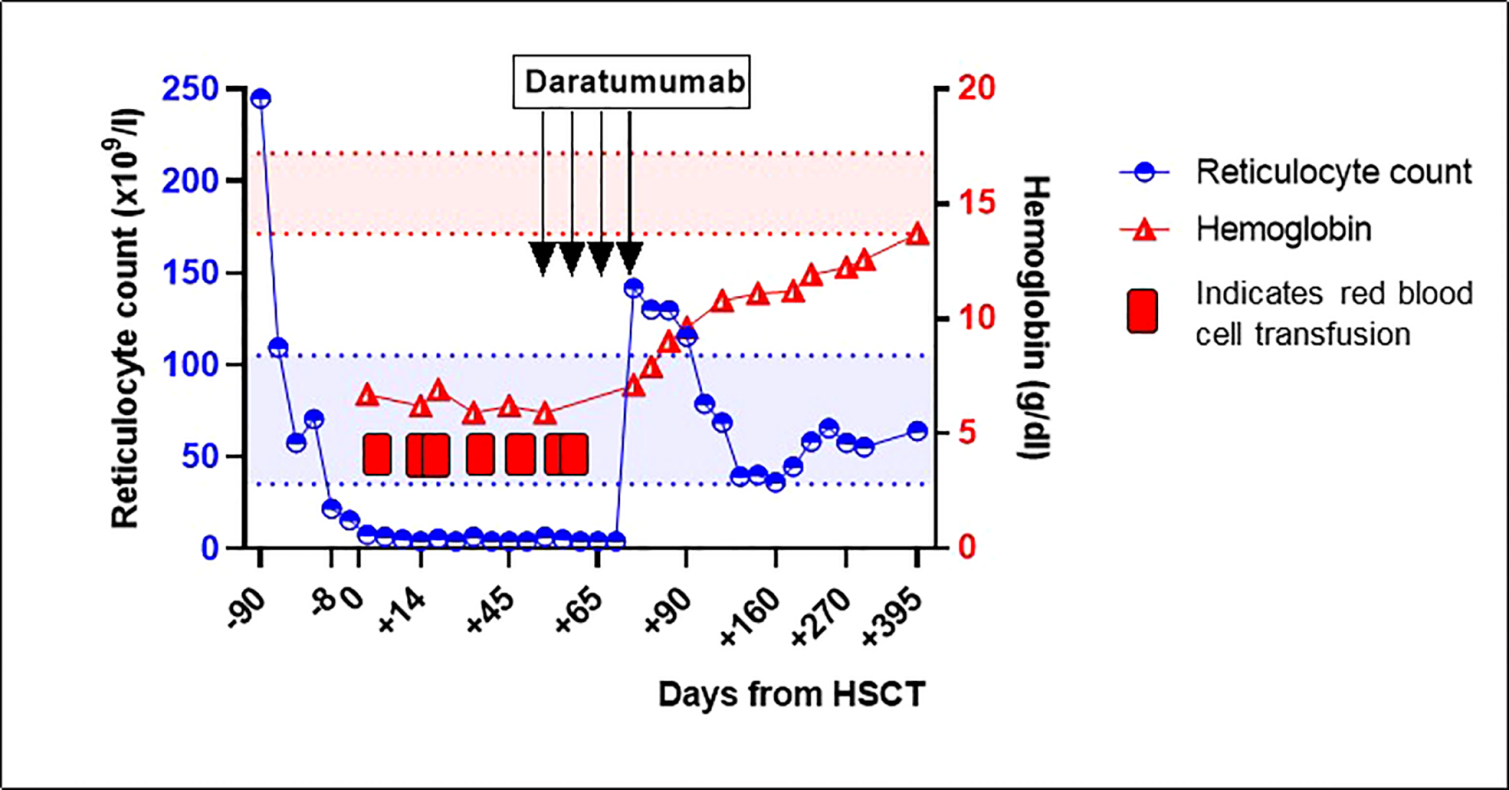
Reticulocyte count and hemoglobin around hematopoietic stem cell transplantation and after treatment with daratumumab for pure red cell aplasia. Between February 2022 and April 2022, the patient received several red blood cell transfusions. The red and blue areas between the dotted lines represent the normal values of reticulocyte count and hemoglobin, respectively. HSCT, hematopoietic stem cell transplantation.

In February 2022, a 27-year old male with SCD was transplanted with an ABO-mismatched HLA-identical sibling donor (patient O positive, donor B positive). He received alemtuzumab 1 mg/kg total dose and total body irradiation (3 Gy) as conditioning, followed by peripheral blood stem cell infusion. Graft versus host-disease (GvHD) prophylaxis consisted of sirolimus. The conditioning regimen was preceded by a 3 month long preconditioning phase with azathioprine 150 mg/day and hydroxyurea 25 mg/kg/day. In the months before HSCT, the patient developed a serious delayed hemolytic transfusion reaction (DHTR). In the past, the patient was diagnosed with anti-Jk(b) antibodies, which had disappeared at the time of the DHTR. However, new anti-Wr(a) antibodies and nonspecific cold auto-antibodies were detected at the time of DHTR. The patient was successfully treated with prednisolone and immunoglobulins. Subsequently, 3 weeks before a planned pre-transplant RBC exchange transfusion, he was treated with rituximab (375 mg/m2) to reduce the risk of another episode of DHTR. Blood group genotyping of the matched sibling donor was performed, which was positive for Jk(b) and negative for Wr(a). No antibodies to the donor’s RBC antigens were present before the transplantation. After transplantation, rapid engraftment of leukocytes and platelets occurred. However, the patient remained anemic with profound reticulopenia, for which he needed regular RBC transfusions, receiving a total of seven RBC units before resolution of PRCA. Physical examination revealed no abnormalities. Donor myeloid chimerism was >95%, ruling out primary rejection of the allograft. A PCR for parvovirus B19 DNA was negative. Post-allogeneic HSCT isohemagglutinin titers remained present (IgG anti-B 1:32, IgM anti-B 1:8). No other RBC antibodies were detected at the time of the PRCA. Pure red cell aplasia was diagnosed and treatment with 4 weekly doses of daratumumab (1800 mg subcutaneously, fixed dose) was started on day +60 post-transplantation. Daratumumab was tolerated fairly well by the patient, besides an episode of fever grade 2 (CTCAE v.5) and headache grade 1 (CTCAE v.5), which occurred after the second dose. After 4 doses of daratumumab, prompt increases of hemoglobin and reticulocyte counts (**Figure 1**) and the disappearance of anti-B isohemagglutinins were observed. Chimerism analysis 12 months post-transplantation showed stable donor myeloid chimerism (>95%) and 69% donor T-cell chimerism. Peripheral blood B-cell count had reconstituted to normal values (absolute count 0.104x10^9^/L, 21.7% of lymphocytes). In the 12 months follow-up period after initiation of daratumumab to date, no transfusions or other immunosuppressive therapies have been necessary, and anti-B isohemagglutinins remain undetectable.

## Discussion

Pure red cell aplasia after HSCT using ABO-mismatched donors is a well-known complication, but no standard treatment for post-transplant PRCA has yet been established. In case series evaluating the natural cause of PRCA after HSCT, the time to spontaneous RBC engraftment was reported to be at least several months but does not occur in all cases (9, 10). Earlier described treatment strategies included erythropoietin, plasmapheresis, or immunomodulatory therapies (rituximab, bortezomib, corticosteroids, donor lymphocyte infusion (DLI), or early tapering of immunosuppressive drugs) (11, 12). However, none of these strategies have been proven effective in a prospective trial. Furthermore, post-transplant PRCA is often not the only factor to consider. The presence of GvHD or infections might limit the application of DLI or immunosuppressive drugs, respectively.

Since the first case report in 2018, 11 cases of successful use of daratumumab as a treatment for PRCA after HSCT have been published (7, 8). Most of these patients suffered from long-lasting PRCA and were heavily pre-treated. It was hypothesized that by eliminating residual recipient plasma cells with daratumumab, PRCA is cured in the context of complete donor chimerism (8). Despite only case reports being published, current expert opinion suggests that daratumumab might be the most effective treatment for post-transplantation PRCA (8, 11, 12).

After non-myeloablative conditioning regimens in patients with SCD resulting in mixed chimerism and generally very low donor T-cell chimerism in the early post-transplant period, tapering sirolimus is not a safe option due to the increased risk of graft rejection (1, 13). Our patient had already received rituximab shortly before HSCT because of DHTR; thus, no additional effect was expected from another course of rituximab. Two important aspects warranted early treatment of the PRCA as opposed to a wait-and-see strategy. First, our patient needed weekly RBC transfusions and had a history of alloimmunization, putting him at risk of the formation of new alloantibodies. Second, major ABO incompatibility is associated with an increased risk of graft rejection (14, 15). As such an immunologic process involved in PRCA might especially increase the risk of graft rejection in the setting of mixed chimerism, we decided to treat our patient with daratumumab as early as day +60 after transplantation. While our patient’s robust response to daratumumab is in line with the recently published cases, this is, to the best of our knowledge, the first description of successful treatment of PRCA in the setting of mixed chimerism after non-myeloablative HSCT in a patient with SCD.

Currently, the longest documented response after daratumumab treatment is 2.5 years (8). Longer follow-up of our case will be necessary to determine if one course of daratumumab is sufficient in achieving a definitive response for PRCA in patients with mixed chimerism. Twelve months after treatment with daratumumab and after reconstitution of peripheral B cells, our patient has normal hemoglobin values with anti-B isohemagglutinins remaining undetectable.

## Conclusion

In conclusion, we report the first case of PRCA in the setting of mixed chimerism after non-myeloablative HSCT for SCD that was successfully treated with daratumumab. Further study of daratumumab for PRCA is warranted to optimize this approach and to determine its optimal dosing and duration.

## Data availability statement

The original contributions presented in the study are included in the article/supplementary material. Further inquiries can be directed to the corresponding author.

## Ethics statement

Written informed consent was obtained from the individual(s) for the publication of any potentially identifiable images or data included in this article.

## Author contributions

ED and EN wrote the manuscript. BB critically reviewed the manuscript. All authors contributed to the article and approved the submitted version.
